# Diagnostic Accuracy of 2 cm Versus 4 cm Insertion Depth for Nasal Swabs for SARS-CoV-2 Rapid Antigen Testing—A Randomized Controlled Trial †

**DOI:** 10.3390/diagnostics16081225

**Published:** 2026-04-20

**Authors:** Rasmus Eið Callesen, Tobias Todsen, Rebekka Consuelo Eið, Sabrina Dandanell Stange, Tobias Gredal, Nikolai Kirkby, Michael Papesch, Christian von Buchwald, Kathrine K. Jakobsen

**Affiliations:** 1 Department of Otorhinolaryngology, Head and Neck Surgery and Audiology, Rigshospitalet, Copenhagen University Hospital, Inge Lehmanns Vej 8, 2100 Copenhagen, Denmark; tobias.todsen@regionh.dk (T.T.); kathrine.kronberg.jakobsen@regionh.dk (K.K.J.); 2 DTU Compute, Technical University of Denmark, 2800 Kongens Lyngby, Denmark; 3 Copenhagen Emergency Medical Services, University of Copenhagen, 2100 Copenhagen, Denmark; 4 Department of Clinical Microbiology, Rigshospitalet, Copenhagen University Hospital, 2100 Copenhagen, Denmark; 5 Department of Clinical Medicine, University of Copenhagen, 2100 Copenhagen, Denmark

**Keywords:** testing, COVID-19, ENT perspective, diagnostics

## Abstract

**Background/Objectives**: Optimal specimen collection is essential for accurate diagnostic tests for upper airway infections. Rapid antigen diagnostic tests (RDTs) are commonly used for SARS-CoV-2 testing, yet the optimal sampling depth remains unclear. This study aimed to compare the diagnostic sensitivity and patient discomfort associated with two nasal swab depths: 2 cm (anterior nasal) and 4 cm (proposed mid-turbinate). **Methods**: In this randomized, paired clinical trial conducted at a public COVID-19 test center in Copenhagen, Denmark, 309 adults presenting for SARS-CoV-2 RT-PCR testing were enrolled. Each participant underwent bilateral nasal sampling using RDTs: one nostril with a 2 cm swab and the other with a 4 cm swab, randomized by side. RT-PCR from oropharyngeal swabs served as the reference standard. Discomfort was rated using a 10-point visual analog scale (VAS). **Results**: Among the 309 participants, 57 (18.4%) tested positive for SARS-CoV-2 by RT-PCR. RDT sensitivity was 62.1% (95% CI: 48.4–74.5%) for 2 cm swabs and 70.2% (95% CI: 56.6–81.6%) for 4 cm swabs, a non-significant difference (*p* = 0.34). Among symptomatic individuals, sensitivity increased to 74.4% (2 cm) and 86.0% (4 cm), though the difference also remained non-significant (*p* = 0.17). Discomfort scores were significantly higher for the 4 cm swab (mean VAS: 5.2) compared to 2 cm (mean VAS: 3.8; *p* < 0.001). **Conclusions**: While not statistically significant, deeper mid-turbinate swabbing (4 cm) showed higher diagnostic sensitivity than anterior nasal swabbing (2 cm), especially in symptomatic individuals. However, this came at the cost of increased discomfort. These findings highlight the importance of balancing diagnostic performance and patient tolerability in pandemic testing strategies. The study contributes valuable evidence to inform future guideline development, particularly regarding swab technique, test accuracy, and feasibility in clinical and public health settings.

## 1. Introduction

**Figure 1:**
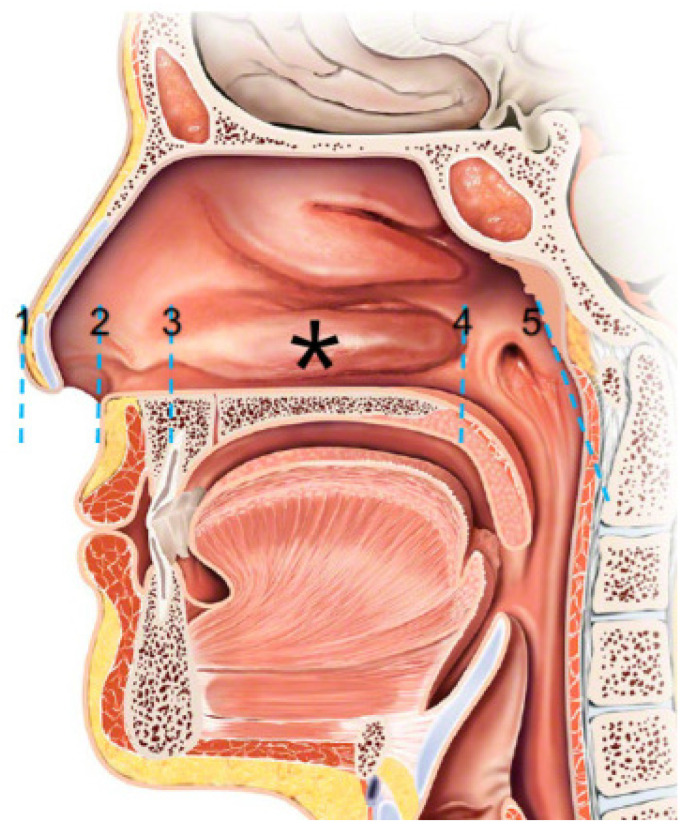
Anatomical landmarks: 1: Tip of the nose. 2: Nasal vestibulum. 3: Anterior part of the inferior turbinate. *: The calculated mid-turbinate insertion depth. 4: Posterior part of the inferior turbinate. 5: Posterior nasopharyngeal wall. Anterior nasal swabs are likely to only test between landmarks 1 and 3, whereas 4 cm will test around the * and should encounter the inferior turbinate.

The global COVID-19 pandemic has underscored the pivotal role of diagnostic testing in infectious disease control [1]. SARS-CoV2 testing is crucial to identify infectious individuals to stop the spread of the disease and start early treatment of individuals at risk of severe COVID-19, as other testing will be for future viruses as well. Rapid diagnostic antigen tests (RDTs) are widely used as cost-effective tests to identify individuals infected with SARS-CoV-2 and reduce transmission [2]. Nasopharyngeal swabs can be challenging to perform correctly, and mid-turbinate swabs and anterior nasal swabs are increasingly used as a minimally invasive alternative for testing [3,4]. Despite the expanded use during the pandemic, the optimal collection method is unclear, and mid-turbinate and anterior nasal swabs are used interchangeably [5]. An anatomical study demonstrated that a nasal swab should be inserted a minimum of 4 cm into the nasal cavity to reach the inferior concha [6]. However, most RDT manufacturers recommend insertion depths of only 2 cm, which will only swab the nasal septum and not reach the inferior concha in most cases (see Figure 1).

The clinical impact of this discrepancy in sampling depth on diagnostic performance has not been thoroughly investigated [7]. Proper specimen collection is the most important step in the diagnosis of infectious diseases, according to [8,9,10].

The main difference between the two sample techniques is the depth to which the swabs are inserted. The anterior nasal swab is to be inserted 1–1.5 cm to collect material from the nasal wall, while the mid-turbinate swab is inserted around 2 cm to sample the inferior concha [8]. In systematic reviews, the anterior nasal and mid-turbinate swabs are often grouped as “nasal swabs”, with a sensitivity ranging from 77% to 93% compared with nasopharyngeal swabs [11]. In a prior study, we recommended inserting the swab approximately 4 cm to ensure a sample from the turbinate [6]. However, the difference in diagnostic sensitivity between our definition of a mid-turbinate swab at 4 cm and what is referred to as mid-turbinate in other studies at 2 cm (which one could argue is just anterior nasal specimen collection) has not been properly explored.

## 2. Materials and Method

### 2.1. Study Design

We conducted an investigator-initiated, randomized clinical trial at a public COVID-19 test center in Copenhagen, Denmark, from 1 February 2023 to 26 February 2023. SARS-CoV-2 testing of oropharyngeal swab specimens with RT-PCR was accessible at testing centers at no cost and without the need for a referral. The Regional Ethics Committee of the Capital Region of Denmark assessed the protocol and exempted it from further processing (protocol no. 21028765). The study was approved by the Danish Protective Agency (protocol no: P-2023-89). We followed the STARD guidelines, and all participants gave verbal and written informed consent before enrollment.

### 2.2. Participants

Citizens, aged 16 years or older, showing up for a SARS-CoV-2 RT-PCR test at Testcenter Valby in Copenhagen, Denmark, were invited to participate in the study on a volunteer basis. In Denmark, SARS-CoV-2 testing during the COVID-19 pandemic was widely available through publicly funded test centers. These centers were established as part of the national testing strategy and offered free testing to the general population without referral. Individuals could book an appointment through the national digital health platform, although walk-in testing was also available. Participants, therefore, represented the general population with no symptoms or with non-characteristic COVID-19 symptoms and were not evaluated by a doctor prior to testing. Patients referred to COVID-19 testing specifically by a doctor with symptoms of COVID-19 were tested in a separate section of the test centers and were not included in this study [12]. In Denmark, citizens were encouraged by the government during the present re-opening-of-society phase to be tested frequently, i.e., every 72 h, hence the implementation of the Ag-test as part of the screening program to enhance test capacity. The exclusion criteria were citizens non-fluent in Danish, and citizens with nasopharyngeal or oropharyngeal anomalies (e.g., neck breathers with tracheostomy), disallowing sampling using swabs. After the enrollment, the participants were surveyed about their symptoms and vaccination status. Because the participants were only mildly symptomatic, none of them needed treatment and were tested to follow the current recommendations for the public or before attending a social event, and none of them needed further medical treatment at the time.

### 2.3. Upper Respiratory Specimen Collection

The enrolled participants were randomized in a 1:1 ratio to have a swab inserted 2 cm in one nostril and 4 cm in the other nostril. First, a healthcare worker performed a throat swab for RT-PCR analysis on all participants to ensure the correct diagnosis (gold standard). Depending on the randomization, either a 2 cm swab was performed in the left nostril and a 4 cm swab in the right, or vice versa.

### 2.4. RDT and Molecular Testing for SARS-CoV-2

A nasal swab from the left and the right nasal cavity was analyzed on separate lateral-flow assays (Standard Q COVID-19 Ag-test, SD Biosensor INC., Suwon, Republic of Korea) for each nasal swab. The healthcare workers were certified in the use and handling of RDTs according to the manufacturer’s instructions. The residual material from the nasal swabs was RT-PCR tested on the STANDARD M10 Point-of-Care MDx system SARS-CoV-2 (SD Biosensor INC.). The swabs for RT-qPCR were collected with a sterile Oropharyngeal Collection Swab (Wuxi NEST Biotechnology Co., Ltd., Wuxi City, China) with a 22 mm swab head of flocked nylon and 150 mm shaft of ABS. The swab heads with specimens were stored in separate sterile tubes with 2 mL of inactivation transport medium (Wuxi NEST Biotechnology Co., Ltd., Wuxi City, China). The samples were sent for RT-qPCR testing at the Serum Institute of the State (Statens Serum Institut/SSI) and the Department of Clinical Microbiology, Rigshospitalet for SARS CoV-2 RT-PCR testing.

### 2.5. Outcome

The primary outcome was to investigate the difference in diagnostic accuracy of antigen-detecting rapid diagnostic tests (Ag-RDTs) for SARS-CoV-2 using mid-turbinate (4 cm) versus anterior nasal (2 cm) specimens. Secondary outcomes included diagnostic accuracy correlated to self-reported symptoms of disease, and evaluation of perceived test discomfort, rated on a 10-point visual analog scale, VAS [13].

### 2.6. Statistical Analysis

A study participant was considered to have a SARS-CoV-2 infection if one or more upper respiratory samples were RT–PCR positive (cycle threshold value < 38 is the gold standard). To compare the 2 cm versus 4 cm nasal swab technique, the sensitivity and specificity of the tests were calculated. The diagnostic accuracy of the samples from these locations was compared to the diagnostic accuracy of samples collected from nasal and throat specimens evaluated by RT-PCR. A test would be considered a false positive if at least one of the RT-PCRs from the same test subject was not positive and would be discarded. A logistic regression analysis using Generalized Estimating Equations (GEEs) was used to compare the rate of RT-PCR positive SARS-CoV-2 detection with RDT in 2 cm versus 4 cm nasal swab technique samples. We defined a level of statistical significance as a *p*-value < 0.05. Data was analyzed in RStudio, version 2024.04.2 + 764.

### 2.7. Invalid Test Results

Invalid test results from either the RT-PCR analysis or the RDTs from a patient were excluded from the respective data analysis. A sample was considered invalid if test results were missing or a control line was missing on the RDT.

### 2.8. Statistical Power

The study was designed as a superiority analysis comparing the diagnostic sensitivity of two RDTs using a paired design. The sample size calculation was based on an anticipated 30% absolute improvement in sensitivity, providing 80% power at a 5% significance level (α = 0.05).

## 3. Results

**Figure 2:**
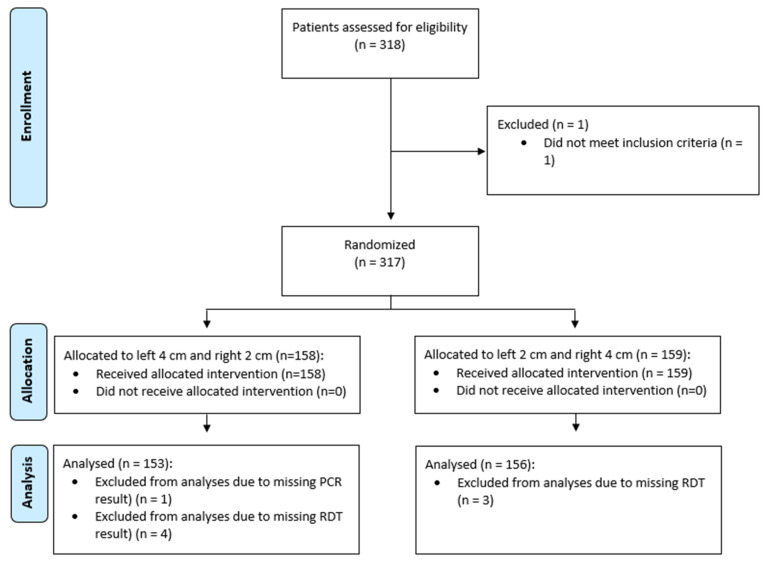
Flowchart.

We enrolled a total of 317 participants (Figure 2); however, eight participants were excluded due to missing test results (either a missing PCR result or both RDT results were unavailable). Consequently, 309 participants were included in the final analysis.

A slight majority were male (57.6%), and the median age at diagnosis was 43 years (IQR: 30–84). Most participants were vaccinated (85.6%), and a large proportion previously had a COVID-19 infection (79.0%) (Table 1).

The most common reason for visiting the test center was the presence of symptoms (58.3%). An additional 23.6% attended either because they believed they had been exposed to COVID-19 or wished to avoid exposing others during social interactions.

Among symptomatic participants, the most frequently reported symptoms were cough (70.7%), sore throat (61.9%), fatigue (61.3%), and headache (57.5%) (Table 1). Only one participant reported symptoms for one day or less, while the majority had experienced symptoms for 4–6 days prior to testing (47%).

### 3.1. Diagnostic Performance

**Figure 3:**
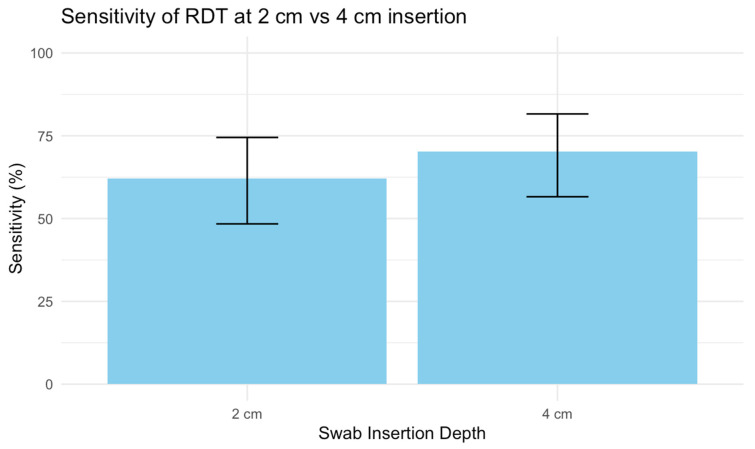
Sensitivity of RDT.

Four participants were missing both RDTs and were excluded from the analysis. Among the remaining 309 individuals, 57 tested positive by RT-PCR, corresponding to a prevalence of 18.4%. When using the RDT at a sampling depth of 2 cm, 36 participants tested positive, compared to 40 participants when the RDT was applied at a depth of 4 cm. This revealed a sensitivity of 62.1% (95% CI: 48.4% to 74.5%) for 2 cm compared to a sensitivity of 70.2% (95% CI: 56.6% to 81.6%) for 4 cm (Figure 3).

A total of 309 participants were enrolled, of whom 57 were confirmed to be SARS-CoV-2-positive, yielding a disease prevalence of 18.4%. The observed difference in diagnostic sensitivity between the two insertion lengths was smaller than expected (8.1 percent points) and did not reach statistical significance (approximate two-sided *p* ≈ 0.34). Nevertheless, the study provides important prospective data on RDT performance in this population.

Using GEE and adjusting for randomization, we found no statistically significant difference in diagnostic performance between the two sampling depths, but with more tests, this would likely become significant (*p* = 0.34).

Among symptomatic participants, the sensitivity of the RDT increased to 74.4% (95% CI: 58.8–86.5%) for the 2 cm sampling depth and 86.0% (95% CI: 72.1–94.7%) for the 4 cm depth. However, the difference in diagnostic accuracy between the two sampling depths remained statistically non-significant when analyzed using GEE, adjusting for randomization (*p* = 0.17). We performed a subgroup analysis stratified by duration of symptoms (<3 days vs. ≥3 days). Among patients with symptoms for less than 3 days, the sensitivity was 0.76 (95% CI: 0.56–0.90) for the 2 cm threshold and 0.86 (95% CI: 0.68–0.96) for the 4 cm threshold. In patients with symptom duration of 3 days or more, the sensitivity was 0.70 (95% CI: 0.35–0.93) for the 2 cm threshold and 0.90 (95% CI: 0.55–1.00) for the 4 cm threshold. When the duration of symptoms was included as a covariate in the GEE model, it was not significantly associated with the outcome.

### 3.2. Vaccination

Among the 256 vaccinated participants, 195 (76.2%) had a prior COVID-19 infection. Overall, 155 (60.5%) reported symptoms, and 48 (18.8%) tested positive. Among symptomatic vaccinated participants, 36 of 155 (23.2%) tested positive. Among the 43 unvaccinated participants, 38 (88.4%) had a prior COVID-19 infection. Twenty-one (48.8%) reported symptoms at the time of testing, and 8 (18.6%) tested positive. Among symptomatic unvaccinated participants, seven of twenty-one (33.3%) tested positive.

### 3.3. Discomfort

**Figure 4:**
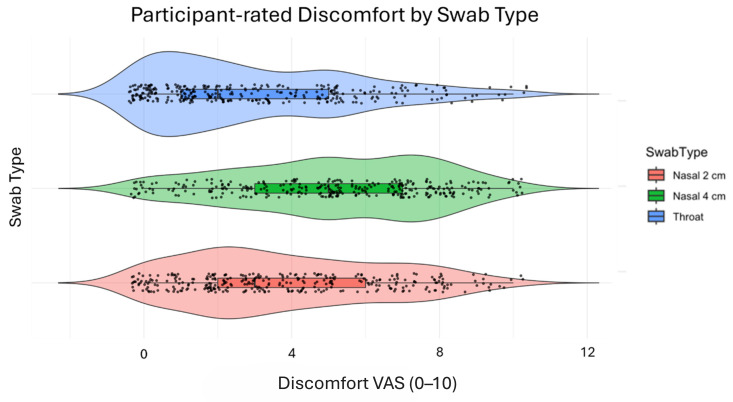
Discomfort. Black dots represent individual participant ratings of discomfort on the visual analogue scale (VAS), illustrating the distribution and density of observations within each swab type.

Participants rated discomfort using a 10-point VAS. Throat swabs were rated least uncomfortable (mean score: 3.0), though seven individuals rated the discomfort as 10/10. Nasal swabbing at 2 cm yielded a mean score of 3.8, while swabbing at 4 cm was associated with the highest discomfort (mean: 5.2, *p* < 0.001). Ten participants rated the 4 cm swab as 10/10 in discomfort; five did so for the 2 cm swab. This can be seen in Figure 4.

## 4. Discussion

In this randomized diagnostic study, we compared the diagnostic sensitivity of the anterior nasal (what CDC calls mid-turbinate) at 2 cm with the diagnostic sensitivity of our suggestion for mid-turbinate at 4 cm. Both were performed using swabs designed for sampling SARS-CoV-2 using RDTs. Although the mid-turbinate swab demonstrated a higher sensitivity than the anterior nasal swab, particularly among symptomatic individuals, the difference was not statistically significant. The discomfort we found in this study is comparable with other studies [13,14,15]. Our RDT nasal tests were rated at 3.8/10 for 2 cm and 5.2/10 for 4 cm, indicating the mid-turbinate is closer in discomfort score to nasopharyngeal wall testing, while a 2 cm swab is closer to that of a throat swab. Based on our results, we cannot disregard testing at 2 cm, given how much more comfort and compliance this could give in the general population. In large-scale screening programs, though, even modest improvements in sensitivity may translate into a substantial number of additional detected cases, potentially reducing further transmission. This is especially important in high-prevalence settings or during outbreaks involving highly transmissible variants, like with the COVID-19 Omicron variant [16]. The strength of our study lies in its randomized, paired design. This reduces inter-individual variability and allows for direct comparison between sampling methods. Furthermore, all tests were administered by trained healthcare workers in a real-world clinical setting, enhancing the generalizability of the findings.

This study has several limitations. The relatively low number of RT-PCR-positive participants limited the statistical power to detect small differences in sensitivity, as well as a risk of type II error due to insufficient sample size. Additionally, while staff were trained in a consistent swabbing technique, individual anatomical differences may have led to variability in the actual sampling site. Finally, our findings may not directly apply to self-administered tests, which remain common in real-world settings. From a public health perspective, the ease and tolerability of anterior nasal swabs make them attractive for mass testing, especially when testing asymptomatic individuals or testing children [17]. However, in high-risk or symptomatic populations where diagnostic sensitivity is critical, the use of a mid-turbinate swab may offer improved detection and should be considered, especially in clinical or point-of-care settings. Overall, this is in accordance with other studies comparing self-collected vs. healthcare worker-collected swab specimens [18,19].

Considering the substantial efforts made to vaccinate a large proportion of the population early in the pandemic, we found it relevant to examine vaccine efficacy within our cohort. The overall positivity rate was similar between vaccinated and unvaccinated participants (18.8% vs. 18.6%). However, among symptomatic individuals, unvaccinated participants exhibited a higher proportion of positive tests (33.3% vs. 23.2%), suggesting a potential protective effect of vaccination against symptomatic infection. Interpretation of these findings is limited by the relatively small number of unvaccinated participants and the high prevalence of prior COVID-19 infection in both groups, which may confound the observed associations. Nevertheless, our results are consistent with previous studies demonstrating that, although vaccinated individuals may still acquire infection (particularly with variants like Delta and Omicron, for example), vaccination remains highly effective in reducing the risk of severe outcomes, including hospitalization and death [16,20].

A study tested patients who had COVID-19 for less than 8 days of symptoms and more than 18 days of symptoms to see if there was a difference in their immune response and final immunity. They observed that patients who had COVID-19 symptoms for more than 18 days exhibited the same levels of SARS-CoV-2-specific cellular immunity as individuals who resolved their symptoms within 8 days [21]. In our subgroup analysis, sensitivity estimates were broadly comparable across symptom-duration groups, although the 4 cm threshold showed consistently higher sensitivity than the 2 cm threshold. However, when we included symptom duration as a covariate in the GEE model, it was not significantly associated with the outcome, so the diagnostic performance of the thresholds cannot be said to be meaningfully influenced by time since symptom onset. These findings indicate that testing is likely to perform similarly in patients presenting with symptoms early and late. One should also be mindful that the wide confidence intervals should be taken into consideration when interpreting these results. In a larger comparative study, participants were stratified by symptom duration, with one group experiencing symptoms for more than 8 days and another for 1–4 days. The authors reported a statistically significant difference in diagnostic sensitivity between these groups, with samples obtained at ≥8 days after symptom onset demonstrating lower sensitivity compared to those collected earlier (1–4 days) (*p* < 0.01) [22]. It is possible that if we had succeeded in including more participants with symptoms for 8+ days and compared those to our group of 2–3 days, we would have found a significant difference in sensitivity.

In a societal setting, when deciding on guidelines, we found it interesting that viral loads in other studies appear to be similar between asymptomatic and symptomatic patients [23]. This means that the transmission potential of the virus can still be high in asymptomatic carriers. In our cohort, although sensitivity was higher among symptomatic participants, both sampling depths demonstrated only moderate diagnostic performance overall. This supports the use of accessible and tolerable methods, such as anterior nasal swabbing, in large-scale screening, while deeper sampling may be reserved for symptomatic or higher-risk individuals where improved sensitivity is desirable. The same study also found that the viral load of SARS-CoV-2 peaks from upper respiratory tract samples around the time of symptom onset or a few days after and becomes undetectable within about two weeks. However, some studies report that for lower respiratory tract samples, this peak may occur at a slightly later stage and that the virus may persist for longer [23]. This is especially relevant for more symptomatic patients enrolled in hospitals, where quick tests do not cut it as a test method anymore.

Regarding our results showing that negative-RT-PCR participants have more smell alterations, we chose to compare with other studies. In a study of 1613 participants in California, they saw that the vaccinated population had more symptoms of congestion, but less fever and myalgia [24]. A large portion of our participants were vaccinated and could perhaps have presented with more congestion symptoms, which could have led to alterations in smell. It is also likely that after initial fever, patients with myalgia and congestion recover from these symptoms, but the smell and taste alterations persist for longer. A meta-analysis of over 13,700 patients shows that the mean for recovery of smell is 12.9 days [25], and an Italian study found that under half (46.8%) of the included patients had regained their sense of smell after 10 days and that 15% had not recovered even after 45 days [26].

Instead of performing mid-turbinate swabs at 4 cm, another test strategy could be to combine anterior nasal swabs with throat swabs to improve the sensitivity of testing. A study found that throat swabs had higher sensitivity for RDT than nasal swabs, and a combination of throat and nasal specimen collection would increase sensitivity by 20% [18]. As throat swabs also have a lower discomfort level than the 4 cm nasal swab [13], a better testing strategy might therefore be to combine a 2 cm nasal swab with a throat swab to increase test sensitivity. Several studies have found that an oropharyngeal swab combined with an anterior nasal swab can be used with an equivalent performance to a nasopharyngeal swab (gold standard) [27,28]. Another study in a similar environment to ours even showed that self-collected combined nasal and oropharyngeal swabs provided equivalent performance when compared to healthcare professional-collected oropharyngeal swabs in detecting SARS-CoV-2 [29]. However, no RDTs are approved for throat swab use [30], whereas 41 different tests are for nasal specimen collection. Throat specimen collection still needs to be a priority for the medical industry to obtain approval for a new test indication, being self-collected. The high sensitivity of throat specimens has also been confirmed for the influenza virus, so throat swabs should be considered for this indication as well [11,31].

Given that viral load varies over the course of infection, an optimal testing strategy for future viral pandemics resembling COVID-19 could involve rapid, accessible tests for mildly symptomatic individuals within the first 1–4 days of symptom onset (potentially self-collected), while patients with more pronounced or prolonged symptoms (>4 days) may benefit from deeper sampling via throat or nasopharyngeal swabs performed by a healthcare professional that are sent for RT-PCR to maximize diagnostic accuracy and capture cases that rapid tests might miss.

## 5. Conclusions

In conclusion, we found no statistically significant difference in diagnostic sensitivity between anterior nasal and mid-turbinate swabs for SARS-CoV-2 using RDT. While both methods are viable options for viral testing, mid-turbinate seems to offer higher sensitivity at the cost of discomfort. Designing guidelines for future pandemics requires consideration of many factors. This study contributes to the growing body of knowledge necessary for that task. Alongside elements such as cost, test availability, and the debate between healthcare-administered and self-administered tests, it adds valuable insights, informing future decision-making. Future research should aim to validate these findings in larger cohorts and across diverse populations, potentially including self-testing settings or in comparison to oropharyngeal tests. Standardization of sampling techniques and clearer anatomical guidelines may help optimize test performance without unnecessarily compromising patient comfort. Ultimately, refining upper airway sampling strategies will not only improve SARS-CoV-2 diagnostics but also strengthen how we plan testing for future respiratory pandemics.

## Figures and Tables

**Table 1:** Baseline characteristics of included participants.

	Overall	Golden Standard ^1^	
		Negative *n* = 252	Positive *n* = 57
Age, mean (SD)	44.71 (16.46)	44.61 (16.32)	45.18 (17.22)
Male sex	178 (57.6)	142 (56.3)	36 (63.2)
Vaccinated	256 (85.6)	208 (85.6)	48 (85.7)
Randomization order			
1 (2 cm left and 4 cm right)	156 (50.5)	130 (51.6)	26 (45.6)
2 (4 cm left and 2 cm right)	153 (49.5)	122 (48.4)	31 (54.4)
Prior positive for COVID-19 by PCR	233 (79.0)	197 (82.4)	36 (64.3)
Test reason			
Symptoms	180 (58.3)	138 (54.8)	42 (73.7)
Exposure to COVID-19	43 (13.9)	29 (11.5)	14 (24.6)
Positive quick test	18 (5.8)	2 (0.8)	16 (28.1)
Screening	8 (2.6)	7 (2.8)	1 (1.8)
Test prior to a social event/visit	30 (9.7)	26 (10.3)	4 (7.0)
Other	96 (31.1)	88 (34.9)	8 (14.0)
Symptoms ^2^			
Sore throat	112 (61.9)	85 (62.3)	26 (60.5)
Headache	104 (57.5)	75 (54.3)	29 (67.4)
Cough	128 (70.7)	95 (68.8)	33 (76.7)
Pain in muscles and joint	84 (46.4)	64 (46.4)	20 (45.5)
Fever	80 (44.2)	54 (39.1)	26 (60.5)
Tired	111 (61.3)	86 (62.3)	25 (58.1)
Reduced sense of taste and/or smell	39 (21.5)	31 (22.5)	8 (18.6)
Days since first symptom ^2^			
0–1 day	28 (16.9)	25 (19.7)	3 (7.7)
2–3 days	78 (47.0)	52 (40.9)	26 (66.7)
4–6 days	35 (19.8)	27 (20.1)	8 (18.6)
>6 days	22 (12.4)	20 (14.9)	2 (4.7)
Unknown	15 (8.4)	11 (8.1)	4 (9.3)

^1^ Gold standard: one or more PCR specimens are positive; ^2^ among individuals with symptoms, corresponding to *n* = 178 participants.

## Data Availability

The original contributions presented in this study are included in the article. Further inquiries can be directed to the corresponding author.
